# *Bombyx mori* Nucleopolyhedrovirus *p26* Is Associated with Viral Late Stage Replication

**DOI:** 10.3390/insects12080707

**Published:** 2021-08-06

**Authors:** Jun-Qing Ge, Zhu-Hong Wang, Xi Chen, Hua Chen, Jian Huang

**Affiliations:** 1 Institute of Biotechnology, Fujian Academy of Agricultural Sciences, Wusi Road 247, Fuzhou 350003, China; kobeid@163.com (X.C.); chxyw2018@163.com (H.C.); 2 Department of Entomology, College of Plant Protection, Fujian Agriculture and Forestry University, Shangxiadian Road 15, Fuzhou 350002, China; wzhuhong@126.com (Z.-H.W.); jhuang1234@126.com (J.H.)

**Keywords:** BmNPV, *p26*, expression, subcellular localization, knockdown, replication

## Abstract

**Simple Summary:**

*p26* is conserved among all completely sequenced Lepidoptera baculoviruses, and some baculoviruses even have two copies of *p26* (*p26a* and *p26b*), which suggested that *p26* may have a basic role in the baculovirus infection cycle. *p26* may be transcribed by the host RNA polymerase II in both early and late infection. Here, protein analyses showed that *Bombyx mori* nucleopolyhedrovirus (BmNPV) *p26* levels were very low amounts during the early phases of infection, which then increased and then declined during the late infection phase. Thus, BmNPV *p26* might have functions in both the cytoplasm and nucleus. Previous *p26* knockout study indicated that *p26* may be an auxiliary gene that does not influence key aspects of viral replication or transmission, and RNAi response to *p26* may somewhat regulate viral replication. Therefore, in order to maintain low *p26* expression and measure BmNPV *p26* function, a RNAi-based knockdown method was chosen. The results indicated that high *p26* expression during the middle interval is necessary for late-stage viral replication. Since *p26* is not essential for baculovirus replication and transmission, it would be interesting to investigate whether *p26* is involved in regulating host innate immune response.

**Abstract:**

*Bombyx mori* nucleopolyhedrovirus (BmNPV) *p26* is conserved among all Lepidoptera baculoviruses that have been completely sequenced thus far, and some baculoviruses even have two copies of *p26*, which suggested that *p26* may play an important role in the virus infection cycle. This study aimed to characterize BmNPV *p26*. We found that BmNPV *p26* transcripts were detectable as early as 3 h post-infection (hpi), and the transcript levels rapidly increased starting from 12 hpi. Western blot analysis using an anti-*p26* polyclonal antibody demonstrated that the corresponding protein was also detectable from 6 hpi in BmNPV-infected cell lysates. Immunofluorescence analysis demonstrated that *p26* was mainly dispersed in the infected cell cytoplasm, whereas the over-expressed fusion protein EGFP-p26 also accumulated in the nucleus. These results indicated that *p26* is an early BmNPV gene and has functions both in the cytoplasm and the nucleus. RNAi-based knockdown of *p26* could produce infectious virus and normal-appearing virions but decreased budded virus (BV) production in BmNPV-infected cells at 72 hpi. Moreover, the results of further quantitative PCR (Q-PCR) analysis indicated that the *gp64* and *p74* transcripts levels decreased significantly. These results indicated that BmNPV *p26* may be associated with BmNPV replication during the late infection stage.

## 1. Introduction

Baculovirus genes are expressed via a highly regulated cascade [1]. Genes expressed in the early phase are transcribed by the host RNA polymerase II in the cell nucleus at 0–3 h post-infection (hpi) [2], and the resulting products are required for DNA replication and late gene expression [3,4]. Viral DNA replication marks the transition from early to late gene expression. Late-stage expression genes are transcribed by virus-encoded RNA polymerase [5], and most of them are involved in viral genome replication and budded virus (BV) production [6]. At very late stages, the major matrix protein for mature occluded viruses, *polyhedrin* (*ph*), is hyperexpressed.

The *Bombyx mori* nucleopolyhedrovirus (BmNPV) genome has been completely sequenced; it contains 136 open reading frames (ORFs) encoding predicted proteins that are > 60 amino acids in length [7]. BmNPV *p26* is encoded by ORF113; its homolog in *Autographa californica* nucleopolyhedrovirus (AcMNPV) is encoded by ORF136 and shares a 97% amino acid identity with BmNPV *p26*. *p26* is usually adjacent to an enhancer sequence (*hr5*) in baculovirus genomic loci and possesses an early promoter motif TATAA [8]. AcMNPV *p26* is transcribed by the host RNA polymerase II during both early and late infection stages [9], and its transcript can be detected at 6 hpi via deep sequencing [10], which indicated that AcMNPV *p26* is an early gene [11]. AcMNPV *p26* knockout analysis indicated that *p26* was nonessential for viral replication [12], had no apparent effect on infectious BV and occlusion-derived virion (ODV) timing or production [13], or required proper virion occlusion in the AcMNPV polyhedra [14]. Moreover, BmNPV *p26* knockout virus could also replicate and produce infectious BVs in *B. mori* cell line BmN [15].

In this study, we examined the expression and subcellular localization of BmNPV *p26* as well as its involvement in BV production in virus-infected BmN cells. The results indicated that BmNPV *p26* transcripts could be detected from 3 hpi, whereas *p26* protein was detectable as early as 6 hpi. Immunofluorescence microscopy showed that *p26* was mainly localized in the cytoplasm, whereas over-expressed EGFP-fusion *p26* was localized in both the cytoplasm and nucleus, and was most accumulated in the nucleus. RNAi-based BmNPV *p26* knockdown could produce infectious virus and normal-appearing virions, but resulted in decreased BV production in BmNPV-infected BmN cells at 72 hpi. Taken together, these results indicated that BmNPV *p26* is a baculovirus early gene but associated with viral late infection events.

## 2. Materials and Methods

### 2.1. Cells and Viruses

BmN cells were maintained at 27 °C in TC-100 insect medium supplemented with 10% (v/v) fetal bovine serum (Gibco). The BmNPV (T3 strain) virus was used as the wild-type virus and propagated in BmN cell line.

### 2.2. Computer-Assisted Sequence Analysis

The ExPASy server software (http://www.expasy.org/tools (accessed on 7 February 2017)) was used for predicting *p26* domains, motifs, signal sequences, and post-translational modifications. Protein homologs were compared using BLASTP with updated GenBank/EMBL databases. Multiple sequence alignments were performed on ClustalW software (http://www.ebi.ac.uk/clustalw (accessed on 7 February 2017)) and edited by using GeneDoc software (version 2.04).

### 2.3. Expression of p26 in E. coli and Generation of Anti-p26 Serum

The *p26* ORF was amplified from the BmNPV genome using the primers p26FW and p26RW (with *Bam*HI and *Hind*III sites, respectively). The PCR products were cloned into the expression vector pET-28a (Novagen, Darmstadt, Germany) to generate the plasmid pET-28a-p26. It was then transformed into *E. coli* BL21 cells, which were induced to express the fusion protein His-p26. The His-p26 fusion protein was purified, extracted, and used to produce anti-*p26* serum in rabbits.

### 2.4. RT-PCR

BmNPV-infected BmN cells were collected at 1, 3, 6, 12, 24, 48, and 72 hpi to isolate total RNA using TRIzol reagent (Invitrogen) according to the manufacturer’s instructions. The first cDNA strand was synthesized by using an oligo-p(dT)18 primer and AMV Reverse Transcriptase (TaKaRa, Dalian, China). Subsequently, *p26*, *p74,* and *ie1* were partially amplified via PCR by using the primer pairs Qp26FW/Qp26RW, Qp74FW/Qp74RW, and Qie1FW/Qie1RW, respectively. The PCR products were analyzed on a 2% agarose gel. *gapdh* was also partially amplified by using the primer pair QBmGapdhFW/QBmGapdhRW and used as the RT-PCR control. The sequences of the primers are included in Table 1.

### 2.5. Immunodetection of p26

Protein samples were prepared from BmNPV-infected BmN cells harvested at 0, 3, 6, 12, 24, 36, 48, and 72 hpi and same number of lysate cell-equivalents were loaded for SDS-PAGE separation. The protein samples were then transferred onto PVDF membrane (Millipore) using a semi-dry Trans-Blot Cell apparatus (Bio-Rad). The rabbit-derived anti-*p26* polyclonal antibody and horseradish peroxidase (HRP)-conjugated goat anti-rabbit IgG were used as the primary and secondary antibodies, respectively. The signal was developed with H_2_O_2_ and diaminobenzidine (DAB) as a chromogenic substrate. The BmNPV *ORF122* protein (*Bm122*) [16], a BmNPV early gene product, was also detected from the collected protein sample series and used as reference control.

### 2.6. Immunofluorescence Microscopy

In order to detect *p26* subcellular localization, BmNPV-infected BmN cells were subjected to confocal microscopy [17]. At 48 hpi, the cells were collected, fixed, and incubated with the anti-*p26* polyclonal antibody or the pre-immune antiserum in 1× PBS for 2 h. The primary antibody was removed and incubated with protein G-fused enhanced green fluorescence protein (EGFP) for 2 h and with the nucleus (DNA)-specific stain DAPI (Sigma-Aldrich, Shanghai, China) for 1 h. Subsequently, the cells were directly visualized and photographed on a Zeiss LSM 510 confocal laser scanning microscope.

### 2.7. EGFP-p26 Over-Expression in BmN Cells

In order to observe potential nuclear localization of *p26*, EGFP-p26 was expressed in BmN cells using the Bac-to-Bac expression system. The recombinant donor vector pFastBacHTb (Invitrogen, Shanghai, China) was reconstructed to contain *egfp* and *p26*, designated as pBacHT-EGFP-p26. The plasmid was transformed into BmDH10Bac *E. coli* (Invitrogen) to generate the recombinant bacmid, designated as EGFP-p26/rBmBac. The extracted EGFP-p26/rBmBac DNA was transfected into BmN cells by using Cellfectin II (Invitrogen). The recombinant virus was obtained and used to infect BmN cells, and fluorescence was directly viewed under a confocal laser scanning microscope at 24 and 48 hpi. EGFP/rBmBac-infected cells were used as the control.

### 2.8. RNAi-Based Knockdown

The primer pair T7p26iFW/T7p26iRW with T7 RNA polymerase promoter sequences at both ends were designed. Partial BmNPV *p26* sequences were then amplified from the BmNPV bacmid. Moreover, *gfp* was amplified by the primer pair T7GFPiFW/T7GFPiRW. The primers used here are included in Table 1. The PCR products were purified and used as templates in order to generate dsRNA by using the MEGAscript dsRNA Kit (Ambion, Shanghai, China), according to the manufacturer’s instructions. The synthesized *p26* dsRNA (dsP26) and *gfp* dsRNA (dsGFP) were purified by using the MEGAclear Transcription Clean-Up Kit (Ambion).

BmN cells were cultured in six-well plates and infected with BVs of BmNPV (T3) (MOI = 1.0). At 3 hpi, the cells were washed and transfected with 5 μg each of dsP26 and dsGFP by using Lipofectamine 3000 (Invitrogen), and non-dsRNA-treated BmN cells were used as the controls. At 24, 48, and 72 h after dsRNA treatment, the cellular supernatants were collected for TCID_50_ titration analysis.

### 2.9. Verification of p26 Knockdown

Total RNA extraction and reverse transcription were conducted as described above, and cDNA synthesized from BmNPV-infected BmN cells treated with dsRNA was used as templates to analyze the *p26* transcript levels via quantitative PCR (Q-PCR) using the primer pair Qp26FW/Qp26RW. Q-PCR was performed on the ABI 7500 real-time PCR system by using SYBR premix Ex Taq (TaKaRa), according to the manufacturer’s protocol. Moreover, *ie1* transcripts amplified by using the primer pair Qie1FW/Qie1RW were included as an internal control. The derived relative quantity (RQ) values were normalized to those of non-dsRNA-treated controls using the ^△△^Ct method. Sequences of the primers are included in Table 1.

### 2.10. Infectious Virus Titration

BmN cells were seeded onto 96-well plates 1 day before titration. BVs from the supernatants of BmNPV-infected BmN cells exposed to dsRNAs were collected, serially diluted, and then added to plates (with 8 wells/sample). After 5 days, the plates were scored for infection by observing cellular cytopathic effects, and TCID_50_/mL was calculated by using the Reed–Muench method.

### 2.11. Electron Microscopy

BmN cells were cultured in six-well plates and infected with BV of BmNPV (T3) (MOI = 1.0). At 3 hpi, the cells were transfected with dsP26 or dsGFP. At 72 h post dsRNA treatment, the cells were harvested for transmission electron microscopy as described previously [18]. Briefly, the cells were fixed with 2.5% glutaraldehyde and then post-fixed in osmium tetroxide, dehydrated in a standard ethanol-acetone series, infiltrated, and embedded in the Spurr medium. Finally, the embedded cell blocks were cut into superthin sections, stained, and viewed under a Hitachi transmission electron microscope.

### 2.12. Transcription of gp64 and p74

In order to further analyze whether *p26* knockdown influences BV or ODV assembly, transcripts of the BV-specific structural protein *gp64* [19] and the ODV-specific structural protein *p74* [20] were analyzed via Q-PCR using the ^△△^Ct method as described above. *gapdh* transcripts were used as the endogenous control [21]. The sequences of the primer pair Qgp64FW/Qgp64RW for *gp64* and Qp74FW/Qp74RW for *p74* are included in Table 1.

## 3. Results

### 3.1. Sequence Analysis of p26

**Figure A1:**
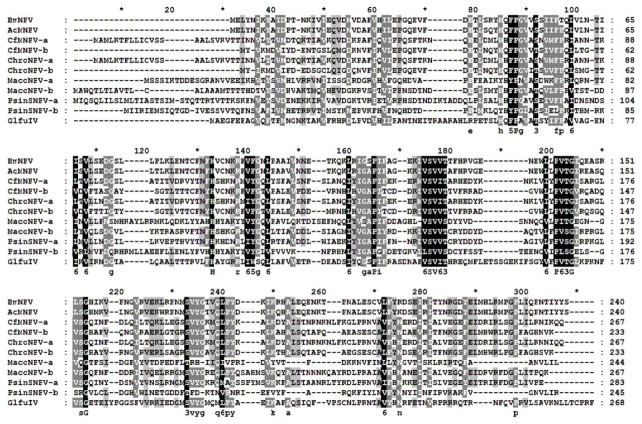
Amino acid sequence alignment of *p26* homologs in *Bombyx mori* nucleopolyhedrovirus (BmNPV, GenBank NP_047534), *Autographa californica* nucleopolyhedrovirus (AcMNPV, GenBank NP_054166), *Choristoneura fumiferana* nucleopolyhedrovirus (CfMNPV-a, GenBank NP_848319; and CfMNPV-b, GenBank NP_848439), *Choristoneura rosaceana* nucleopolyhedrovirus (ChroNPV-a, GenBank YP_008378498; and ChroNPV-b, GenBank YP_008378375), *Mamestra configurata* nucleopolyhedrovirus A (MacoNPV-a, GenBank NP_613192; and MacoNPV-b, GenBank NP_613241), *Pseudoplusia includens* single nucleopolyhedrovirus (PsinSNPV-a, GenBank YP_009116933; and PsinSNPV-b, GenBank YP_009116975), and *Glypta fumiferanae* ichnovirus (GlfuIV, GenBank AKD28026). GeneDoc software was used for homolog shading: black, dark gray, and light gray shading denotes 100%, 80%, and 60% similarity, respectively.

*p26* is 723 nt in length and is located at 107,702–108,422 nt in the BmNPV (T3 strain) genome, which was adjacent to the enhancer sequence (*hr5*) and upstream of *p10*. Some predicted post-translational modification sites and functional motifs of *p26* were explored by using EXPASY tools. However, *p26* does not contain any directed domains or signal peptides that may indicate its function. Here, homologs from other baculoviruses were noted to share 27–98% identity with BmNPV *p26*; interestingly, a structural protein *p26* (GenBank AKD28026) was found in *Glypta fumiferanae* ichnovirus (GlfuIV) with 35% identity (Figure A1). Furthermore, some group I and II alphabaculoviruses were found to have two copies of *p26*, such as *Choristoneura fumiferana* multiple nucleopolyhedrovirus (CfMNPV) [22], *Choristoneura rosaceana* nucleopolyhedrovirus (ChroNPV) [23], *Mamestra configurata* nucleopolyhedrovirus A (MacoNPV) [24], and *Pseudoplusia includens* single nucleopolyhedrovirus (PsinSNPV) [25], suggesting that *p26* may play an important role in the virus infection cycle (Figure A1).

### 3.2. Transcription of p26

**Figure 1:**
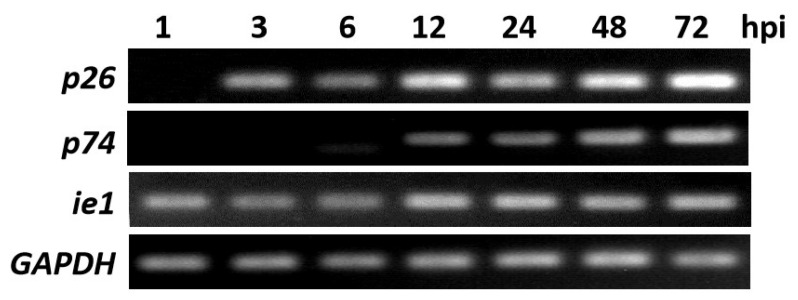
RT-PCR analysis of *p26* transcripts in BmNPV-infected BmN cells. RT-PCR was performed by using purified RNA isolated from virus-infected cells at the indicated time points (1, 3, 6, 12, 24, 48, and 72 hpi). The first cDNA strand was synthesized with AMV Reverse Transcriptase and the oligo-p(dT)18 primer, and nested PCR was then performed with the *p26*, *p74*, and *ie1*-specific primers, respectively. *gapdh* was used as the control.

The temporal transcription of *p26* was examined via RT-PCR by using total RNA isolated from BmNPV-infected BmN cells at different time points. The BmNPV *p26* transcript was detectable as early as 3 hpi, much more abundant from 12 hpi, and continued to be detectable until 72 hpi (Figure 1). Meanwhile, the BmNPV late gene, *p74*, was detectable from 12 hpi, and the early gene, *ie1*, was detectable from 1 hpi. This result indicated that BmNPV *p26* is an early expression gene, which corresponds to the presence of the early transcription start motif [10].

### 3.3. Immunodetection of p26

**Figure 2:**
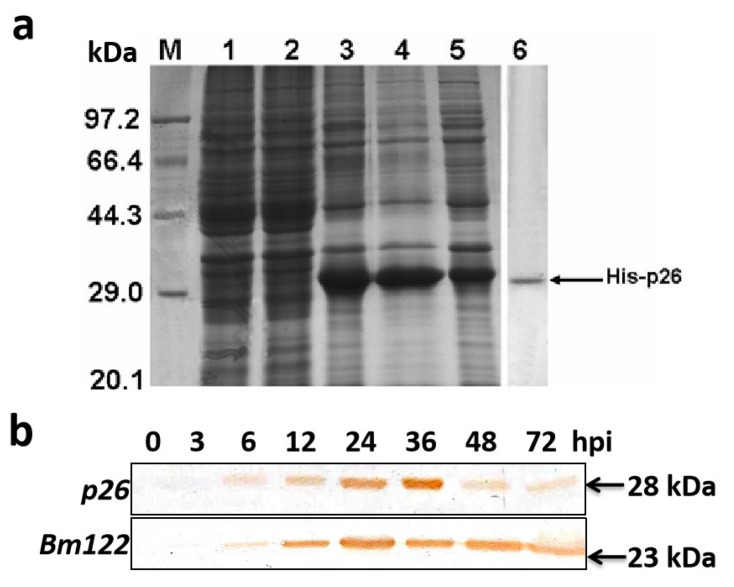
Overexpression of *p26* in *E. coli* and Western blot analysis of *p26* in BmNPV-infected BmN cells. (**a**) SDS-PAGE and Western blot analysis of the fusion protein His-p26 expressed in *E. coli* BL21 cells. M, protein molecular weight markers; Lane 1, BL21 cell proteins; Lane 2, proteins from BL21 cells transformed with pET-28a plasmid; Lane 3, proteins from BL21 cells transformed with pET-28a-p26; Lane 4, proteins in the sediment of supersonically broken BL21 cells transformed with pET-28a-p26; Lane 5, proteins in the supernatant of supersonically broken BL21 cells transformed with pET-28a-p26; Lane 6, Western blot analysis of the expressed His-p26 protein using an anti-His monoclonal antibody. (**b**) BmN cells were infected with wild-type BmNPV (MOI = 10.0), and protein samples were then harvested at 0, 3, 6, 12, 24, 36, 48, and 72 hpi, separated via 10% SDS-PAGE, transferred onto PVDF membranes, reacted with an anti-*p26* polyclonal antibody or an anti-*Bm122* polyclonal antibody, and detected with a DAB substrate. The immunoreactive bands are indicated by arrows, and the size is indicated on the right.

In order to obtain anti-*p26* serum, the *p26* coding region was expressed in *E. coli* (Figure 2a). The expressed fusion protein His-p26 was purified and used to raise polyclonal anti-*p26* serum. Western blot analysis of protein samples extracted from BmNPV-infected BmN cells detected a specific protein of approximately 28 kDa (Figure 2b). The protein was first detected at 6 hpi, and became much more abundant at 36 hpi. However, by 48 and 72 hpi, its levels declined and it became almost undetectable. We had tried to utilize a mouse monoclonal antibody to detect beta-actin as a loading control but failed to detect any positive band. However, another BmNPV early gene, *Bm122*, could maintain high expression levels at 48 and 72 hpi, confirming that BmNPV *p26* had a low expression level in the late stage.

### 3.4. Subcellular Localization of p26

**Figure 3:**
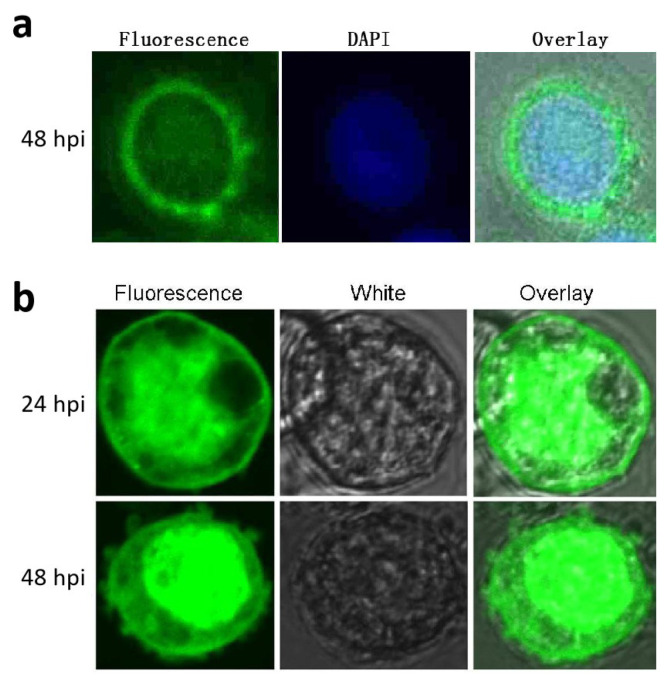
Subcellular localization of BmNPV *p26*. (**a**) Localization of *p26* in BmNPV-infected BmN cells. The cells were collected at 48 hpi, reacted with anti-*p26* serum, treated with EGFP-conjugated goat anti-rabbit IgG, and viewed under a confocal laser fluorescence microscope. In order to visualize the nucleus, the cells were also stained with DAPI (blue). (**b**) Localization of the over-expressed fusion protein EGFP-p26 in recombinant BmNPV infected BmN cells. EGFP-fused *p26* was driven by the AcMNPV *ph* promoter and over-expressed in BmN cells. At 24 and 48 hpi, the cells were directly collected for fluorescence microscopy analysis.

Subcellular localization of *p26* was performed via immunofluorescence analysis. At 48 hpi, BmNPV-infected cells were examined for fluorescence. It was mainly detected in the cytoplasm, and some staining of the nucleus was also observed (Figure 3a). In the controls, no fluorescence was detected when pre-immune serum was used (data not shown), suggesting that *p26* might play a role in both the cytoplasm and the nucleus.

As *p26* were detected in the nucleus via immunofluorescence analysis, in order to more clearly confirm potential nuclear localization of *p26*, EGFP-p26 was over-expressed under the AcMNPV *ph* promoter in BmN cells using the Bac-to-Bac expression system. A recombinant bacmid, EGFP-p26/rBmBac, was extracted and transfected into BmN cells. At 24 and 48 hpi, the cells were directly observed under a confocal laser scanning microscope. The results indicated that the fluorescence still could be observed in the cytoplasm, but it became accumulated in the nucleus (Figure 3b). By contrast, when only EGFP was expressed, uniform fluorescence was observed in both the cytoplasm and the nucleus (data not shown).

### 3.5. Knockdown of p26

**Figure 4:**
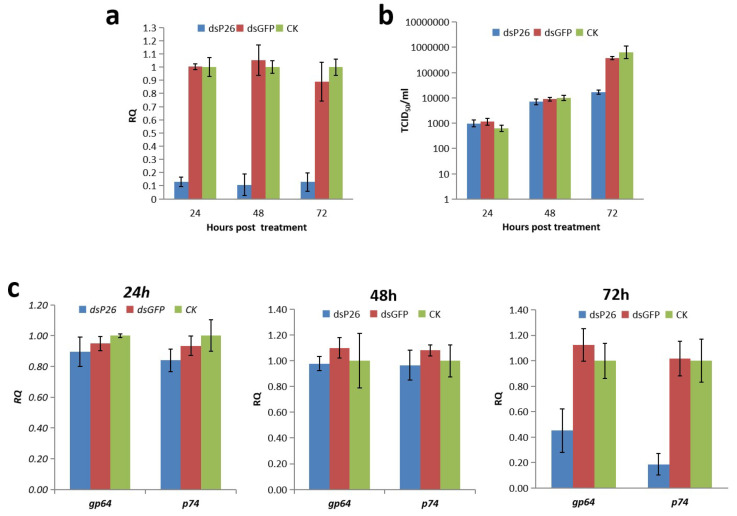
*p26* knockdown by RNAi and its influence on viral replication. BmN cells were infected with BmNPV. At 3 hpi, the cells were transfected with dsP26 and dsGFP, and non-dsRNA-treated BmN cells (CK) were used as the control. (**a**) Q-PCR analysis of *p26* knockdown in BmNPV-infected BmN cells. Total RNA was extracted from BmNPV-infected BmN cells at 24, 48, and 72 h post-dsRNA treatment, reverse transcription and Q-PCR were performed, and the relative *p26* transcript levels were analyzed using the ^△△^Ct method. The derived relative quantity (RQ) values were normalized to those of non-dsRNA-treated controls, and *ie1* transcript detection was included as the internal control. Data are presented as the mean RQs ± SEMs for three replicates. (**b**) Titration of BV from BmNPV-infected BmN cells in which *p26* was knocked down by using dsRNA. At 24, 48, and 72 h post-dsRNA treatment, the supernatants of BmNPV-infected BmN cells were collected for titration by using the TCID_50_/mL method. Data are presented as the mean RQs ± SEMs for three replicates. (**c**) Q-PCR analysis of *gp64* and *p74* transcripts in BmNPV-infected cells with *p26* knockdown. *gapdh* detection was included as internal controls. Data are presented as the mean RQs ± SEMs for three replicates.

Considering that *p26* transcripts were first detectable at 3 hpi, we used this time point for RNAi treatment. Q-PCR analysis indicated that the *p26* transcript level was downregulated by about 90% from 24 to 72 h post-dsRNA treatment (Figure 4a), demonstrating that the synthesized dsP26 could be used to effectively knockdown *p26* transcription

**Figure 5:**
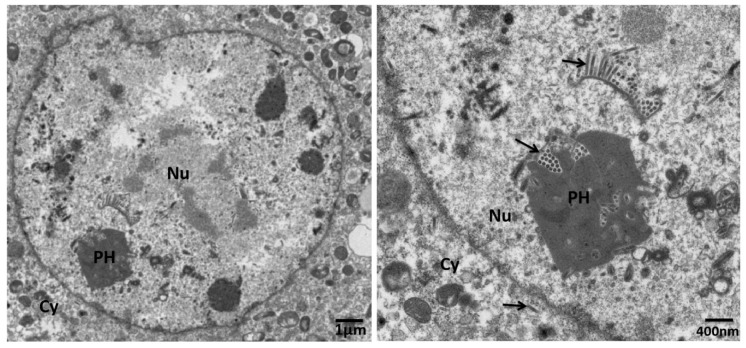
Electron microscope analysis of BmNPV infected BmN cells after *p26* knockdown. At 72 h post-dsRNA treatment, the cells were harvested for transmission electron microscope analysis. Cy, Nu, and PH denote cytoplasm, nucleus, and polyhedra, respectively. The black arrow refers to nucleocapsids and virions.

In order to determine the effects of *p26* knockdown on BVs production, cell culture supernatants of BmNPV-infected cells were harvested for BV titration at the selected time points. BV production from *p26* knockdown cells demonstrated no differences compared with the controls at 24 and 48 h post-dsRNA treatment, but it was significantly reduced at 72 h post-dsRNA treatment (Figure 4b). Furthermore, in order to clarify whether *p26* knockdown influences of BV or ODV assembly, transcripts of BV specific gene *gp64* and ODV specific gene *p74* were detected by Q-PCR. The results showed that both *gp64* and *p74* had expression patterns consistent with above titration results, with no difference at 24 and 48 h post-dsRNA treatment but with a reduction at 72 h post-dsRNA treatment (Figure 4c). However, electron microscopy revealed that *p26* knockdown virus could produce nucleocapsids with normal appearance; enveloped nucleocapsids were noted in the nucleus and cytoplasm, and assembled in the polyhedra (Figure 5).

## 4. Discussion

We used insect cell cultures and RNAi knockdown of *p26* to explore the function of baculovirus genes. Results from this study show that BmNPV *p26* knockdown did not influence BV production at 24 or 48 h post-dsRNA treatment. However, at 72 h post-dsRNA treatment, the BV production titer was significantly reduced. This result indicates that increased *p26* expression during the middle interval of infection is necessary for late-stage viral replication. Even though *p26* is conserved among all sequenced Leipdoptera baculovirus genomes, some baculoviruses are reported to have two copies of *p26* (*p26a* and *p26b*) [25]. The RNAi pathway plays an important role in antiviral responses in insects [26], and the hotspots of siRNA in the HaSNPV genome were detected within *p26*, suggesting that an RNAi response to *p26* may in some way regulate viral replication [10,27]. The two *p26* copies likely have distinct functions [25] and are acquired independently from different sources [22]. Phylogenetic analysis suggests that *p26* was obtained from three independent acquisition events within the baculoviridae family [25]. *p26a* is usually adjacent to *p10*, and *p26b* is usually adjacent to *iap-2* [25]. BmNPV *p26* was noted to be adjacent to *p10*, indicating that it might have the same origin as *p26a*.

In AcMNPV, *p26* has an early promoter with a canonical TATA box, but it lacks a late promoter with a TAAG motif. Nevertheless, it is transcribed by the host RNA polymerase II both early and late in infection [10,28]. MacoNPV *p26a* contains a consensus early promoter, whereas MacoNPV *p26b* has a late promoter; notably, *p26* functions are required during both early and late infection [24]. Sequence analysis results showed that an early transcription TATA box motif was found 24 nt upstream of the putative *p26* initiation codon (ATG). Moreover, *p26* transcripts could be detected at 3 hpi, confirming that *p26* is an early BmNPV gene. Furthermore, its transcription level was investigated via Q-PCR and compared with the early gene *ie1* and late gene *p74*. The results indicated that *p26* had a high transcription level from 12 hpi [10], but no obvious differences were found among *p26*, *ie1,* and *p74* (data not shown).

The BmNPV *p26*-specific antibody detected an approximately 28 kDa protein in BmNPV infected BmN cells, which is an approximation relative to the predicted molecular mass of 27.0 kDa. Protein analyses showed that BmNPV *p26* was detected in very low amounts during the early phase of infection; it later accumulated and then declined in levels during the late infection phase. In the proteomic analyses of AcMNPV ODV proteins, *p26* has not been detected [29], suggesting that *p26* might not be a structural virion protein. In order to investigate whether *p26* is a structural component of BmNPV virions, Western blot analysis of BVs and ODVs was performed by using the *p26*-specific antibody, however, no positive bands were detected (data not shown). Immunofluorescence analysis indicated that BmNPV *p26* was mainly localized in the cytoplasm of infected cells. Nevertheless, weak signals were detected in the nucleus, which is similar to the result noted for AcMPNV *p26* [13,30]. However, the over-expressed fusion protein EGFP-p26 accumulated in the nucleus. Although over-expressed *p26* might have altered its localization, it provided further evidence that *p26* might play a role in both the cytoplasm and the nucleus. Although *p26* does not have a recognizable nuclear localization signal, it was observed to form dimers under physiological conditions [30]; thus, its transport to the nucleus late in infection probably depends on another protein.

AcMNPV *p26* knockout studies have been performed before and indicated that *p26* may be an auxiliary gene that does not influence key aspects of viral replication or transmission [12,13]. For example, the *p26* of a polydnavirus (PDV), *Glypta fumiferanae* ichnovirus (GlfuIV) [31], is transmitted by endoparasitic wasps during egg laying into caterpillar hosts. The main function of PDV is the manipulation of host immunity and improvement of host suitability for the parasitoid [32]. Since *p26* is not essential for baculovirus replication and transmission [13,14], it may provide acceptance for infection by suppressing the insect host innate immune response.

## Figures and Tables

**Table 1:** Primers used in this study.

Name	Sequence	Target
p26FW	5’-CGGATCCATGGAATTGTATAATATTAAAT-3’	*p26*
p26RW	5’-CAAGCTTTTAGCTGTAATATATTGTGTTG-3’	
T7p26iFW	5’-TAATACGACTCACTATAGGGTTTCCTGGCGTCGTTAGTTC-3’	*p26*
T7p26iRW	5’-TAATACGACTCACTATAGGGTTGCACAGTCCCGTAAACAG-3’	
T7GFPiFW	5’-TAATACGACTCACTATAGGGTGGTAAAAGGACAGGGCCATC-3’	*gfp*
T7GFPiRW	5’-TAATACGACTCACTATAGGGCCATGGCCAACACTTGTCAC-3’	
Qp26FW	5’-TGTAATAGAGCAAGTCGACAATGTG-3’	*p26*
Qp26RW	5’-TGGTACCGGCTTAGCGTTTC-3’	
Qie1FW	5’-AACATTTGCACGGTCGCTTC-3’	*ie1*
Qie1RW	5’- GGTCGGAGAACCTGTTGGAA-3’	
Qgp64FW	5’-ACGGCATCAGCAAAAACGTG-3’	*gp64*
Qgp64RW	5’-AAGGTGGACGAGCGTTTGAT-3’	
Qp74FW	5’-TCTGTAGTGGTATCGCGCAC-3’	*p74*
Qp74RW	5’-AGCGCCTTCCAGCATACTAC-3’	
QBmGapdhFW	5’-AGGGCAGTGTTGAGGTTCAG-3’	*gapdh*
QBmGapdhRW	5’-GGCCTTAGGGTCCCTTTCTG-3’
